# Rural Hoarding in Older Adults: A Pilot Characterization Study

**DOI:** 10.1093/geroni/igaa057.987

**Published:** 2020-12-16

**Authors:** Mary Dozier

**Affiliations:** Mississippi State University, Mississippi State, Mississippi, United States

## Abstract

There has been limited research on geriatric hoarding disorder in rural areas. Older adults living in rural areas are more likely to feel stigmatized due to mental health difficulties and to have multiple barriers to healthcare. The purpose of this study is to present the clinical picture of eight older adults (mean age 68, range 57-92) with hoarding disorder who live in the rural southeastern U.S. Participants completed a semi-structured interview and the NIH Toolbox Emotion and Cognition Batteries in their homes. Participants were mostly female (n = 6) and identified as White (n = 5) or African American (n = 3). All participants reported being Christian. Most participants were divorced or had never married (n = 6). All participants reported having at least one current medical condition, with the most commonly reported diagnosis being high blood pressure (n = 4). Half of participants reported that they had experienced at least one intervention from their family; however, only one participant reported ever experiencing an intervention from another source (i.e., property manager). On average, participants reported having a low level of emotional support and life satisfaction and a high level of loneliness and somatic symptoms of fear. Participants’ performance on tests of cognitive functioning was worst for processing speed, with three out of the eight participants performing at the level of borderline impairment or worse. Understanding the clinical presentation of hoarding disorder in rural-dwelling older adults is the first step to the development and implementation of evidenced-based treatments in this population.

